# The Use of the Parents’ Evaluation of Developmental Status and Developmental Milestones in Screening Children for Developmental Delay in India †

**DOI:** 10.3390/children11121530

**Published:** 2024-12-18

**Authors:** Hina Sheel, Lidia Suárez, Nigel V. Marsh

**Affiliations:** 1 Faculty of Health and Life Sciences, De Montfort University Dubai, Dubai, United Arab Emirates; 2 School of Social and Health Sciences, James Cook University, Singapore 387380, Singapore; 3 Tropical Futures Institute, James Cook University, Singapore 387380, Singapore

**Keywords:** developmental delay, typical developmental, PEDS, PEDS:DM, screening, India

## Abstract

Background/Objectives: The Parents’ Evaluation of Developmental Status (PEDS) and Developmental Milestones (PEDS:DM) are cost-effective, self-report tools that can be conveniently utilized in low- and middle-income countries to screen for developmental delays in children. This study assessed the diagnostic accuracy of PEDS and PEDS:DM in distinguishing children with typical development (TD) from those with developmental disabilities (DD). It also examined the relationship between parents’ general concerns and specific developmental concerns about their children. Method: The study included 407 children with TD and 59 children with DD, recruited from diverse socioeconomic backgrounds in Chandigarh, Himachal Pradesh, and the National Capital Region of India. Parents of children aged 4 to 8 years completed the PEDS and PEDS:DM online. Results: The PEDS demonstrated high sensitivity (91%) but low specificity (47%), whereas the PEDS:DM showed poor sensitivity (17%) and specificity (6%). Parents of TD children who expressed general developmental concerns were likely to report specific concerns related to behavior, self-help skills, health, and cognitive development. Parents of DD children with general concerns reported specific issues with fine motor skills, behavior, school performance, cognitive development, and health. Conclusions: These findings suggest that while PEDS and PEDS:DM can support early detection of developmental delays, their interpretation should be approached with caution. The study provides preliminary insights into the applicability of these screening tools for children aged 4–8 years in India.

## 1. Introduction

In India, the population of people identified as differently abled increased from 22 to 29 million from 2001 to 2011. By 2050, it is expected that it will constitute 19% of the population [1]. Developmental screening identifies children who lag behind (children with developmental disability) in developmental milestones compared to typically developing peers (children with typical development) [2,3]. Developmental screening has significant potential to improve the lives of young children and has been utilized worldwide for over two decades [4]. Both screening and surveillance play a crucial role in identifying developmental delays early, ensuring timely intervention to prevent these delays from becoming more severe as the child grows [5]. However, the early identification of developmental and behavioral issues in children is often hindered by limited awareness and inadequate access to screening tools [6].

Both urban and rural schools in India lack the resources necessary to screen students for DD [7]. Physicians frequently rely on unofficial means of early detection, and healthcare authorities are frequently ignorant of screening instruments that might be used to assist parents in understanding their children’s developmental milestones [8,9]. Children with mild-to-moderate emotional and learning disabilities are frequently overlooked as a result of such approaches [6]. Inquiring about parents’ concerns about their child’s growth is one method for early detection. Several studies suggest there may be a direct correlation between the kinds of concerns parents have about their child’s growth and the problems that are later diagnosed [6,9,10]. Parents often make a variety of statements when asked about their child’s development [11], and those words have a high probability of revealing a disability. When interacting with their child, parents might feel concerned about the child’s behavior both at home and in the community. Furthermore, a single parental concern may often mask multiple underlying issues that remain unnoticed by the parents [9].

For children aged 0–8, the Parents’ Evaluation of Developmental Status (PEDS) is a screening and surveillance tool. The tool elicits parents’ concerns about their child’s development, which also helps clinicians decide whether a child needs routine monitoring and early intervention [12]. In addition to estimating developmental risk as high, medium, and low risk, the tool evaluates developmental milestones across clinical samples and communities [13]. Since it more accurately predicts a child’s development than PEDS alone, the Parents’ Evaluation of Developmental Status: Developmental Milestones (PEDS:DM), a tool created after the PEDS, is advised to be used with PEDS [11]. While PEDS:DM assesses the same developmental milestones as PEDS, it offers more age-appropriate items for every age group [14].

In PEDS, parents’ general concern is expressed through their answer to the first question, in which the parent is asked, “Please list any concerns about your child’s learning, development, and behaviour”, and specific concern is expressed by answering the remaining questions of PEDS that specifically focus on specific developmental milestones. For example, “Do you have any concerns about how your child behaves?” is a question related to specific concerns about behavior skills. In addition, PEDS comprises 10 domains. PEDS:DM evaluates children in a similar domain as PEDS. However, for older children, PEDS:DM also assesses reading and math skills. PEDS and PEDS:DM can identify children with DD as compared to children with TD. PEDS has an acceptable sensitivity of 86% and specificity of 74% [8], and PEDS:DM have a sensitivity of 80% and specificity of 85% [14]. PEDS has shown acceptable-to-good sensitivity and specificity in Thailand (sensitivity 67%, specificity 60%) [15] and the United States of America (PEDS, sensitivity 41%, specificity 79%; PEDS:DM, sensitivity 88% and specificity 13%) [16].

In several countries, PEDS and PEDS:DM are used to screen for developmental problems in children. For instance, when applied to a clinical sample, Soucy et al. [17] reported concerns about at least one PEDS domain for the clinical sample. Parents of children with autism spectrum disorder (ASD), specific language impairment (SLI), DD, and TD were given the PEDS form in Australia. Parents of children with ASD had the most significant concerns, according to the data, followed by parents of children with DD, SLI, and TD [18]. Parents of children with ASD completed the Modified Checklist for Autism in Toddlers Revised, which, along with the PEDS, identified developmental issues [19]. Glascoe’s research revealed that parents of children with an IQ below 79 often expressed concerns about behavior, academic writing, speech, and language development [6]. In contrast, Pinto-Martin et al. [20] found that when children were screened for ASD using a tool specifically designed for the condition, developmental concerns were not raised on the PEDS.

The PEDS and PEDS:DM work well in developing countries like India due to their low cost, reliance on self-reporting, and strong psychometric properties [12]. However, research on their use in India remains limited [7]. In India, the PEDS has been used to screen children under the age of five [21,22,23]. These studies reported the diagnostic accuracy of the screening tool to be sub-optimal, with sensitivity (75%) and specificity (74%). However, the screening tool has not been used to screen children aged 4–8 years in India. Furthermore, Malhi and Singhi [21,22] did not use PEDS:DM. Therefore, the first aim of the current study was to explore the sensitivity and specificity of PEDS and PEDS:DM to classify 4- to 8-year-old children with TD and DD in India. Adequate sensitivity is equal to or higher than 80%, and specificity is equal to or higher than 90%. The current study’s analyses were conducted, and the format for presenting the results followed that of Ilić et al.’s [9] study of 289 parents of TD children in Serbia. Ilić et al.’s study aimed to assess the type and level of concerns raised by parents by comparing general and specific concerns. Ilić’s investigation demonstrated a significantly large correlation between generalized and specific concerns pertaining to conduct and socio-emotional functioning. Furthermore, a smaller yet statistically significant correlation was observed between fine motor abilities and expressive language [9]. Their results suggested that when parents report general concerns about their child’s development, those concerns indicate specific developmental issues. These findings are essential to detect children at risk of developmental issues. Therefore, the second aim of the study was to explore whether parents’ general concerns regarding their child’s development, as reported in PEDS, were associated with specific developmental concerns that could suggest developmental issues. In addition, the current study also included an analysis of the reports from parents whose children had DD.

## 2. Method

### 2.1. Research Setting

Researchers conducted a quantitative study in India, where the education system follows the British structure. It includes kindergarten for children aged 2–4, primary school for grades 1–5 (ages 5–11), and middle school for grades 6–8 (ages 11–14) [24]. Schools in India are categorized as either private or public [25].

### 2.2. Participants

Researchers recruited participants using convenience sampling from private inclusive schools in rural and urban areas of Chandigarh, Himachal Pradesh, Punjab, Haryana, and the National Capital Region of India. These inclusive schools serve children with typical development (TD) and developmental delays (DD). Children were classified as having DD based on school records, which included reports from clinicians in government hospitals who had assessed them using standardized tests. Parents submitted these reports to the schools during the admission process. All the states and union territories involved in the study are located in North India, where residents commonly speak Hindi, English, and regional languages [26].

The study initially included parents and teachers of 466 children, with 454 TD children and 61 children with DD. However, data from 47 TD children and 2 DD children were excluded due to missing information or failure to meet the study’s inclusion criteria. To be eligible, participants had to be parents of children aged 4–8 years, Indian citizens, and literate in English or Hindi at least to the level of Primary 6. Parents whose children were not currently attending school were excluded. The final sample consisted of parents and teachers of 407 TD children and 59 DD children.

### 2.3. TD Sample

The TD sample’s parents were 131 (32%) men and 276 (68%) mothers. The parents were between the ages of 23 and 51 (M = 34.75, SD = 5.73). Table 1 shows the parents of the TD sample’s highest level of education and annual household income. Children with TD were between the ages of 4 and 8 (M = 5.81, SD = 1.03). Males made up the majority of the TD sample (*n* = 259, 64%), while females made up 148 (36%) (Table 1).

### 2.4. DD Sample

The DD sample’s parents were 18 (31%) fathers and 41 (69%) mothers. The parents were between the ages of 25 and 51 (M = 35.54, SD = 4.44). Table 1 shows the parents of the DD sample’s highest level of education and annual household income. Children with DD were between the ages of 4 and 8 (M = 4.63, SD = 0.82). In the DD sample, there were 16 (27%) females and 43 (73%) males. The sociodemographic details of the participants (parents) of children with TD and DD are shown in Table 1.

## 3. Measures

### 3.1. Parents’ Evaluation of Developmental Status (PEDS)

The Parents’ Evaluation of Developmental Status (PEDS) [8] is a surveillance and screening tool designed for children aged 0–8 years to identify and address developmental, behavioral, and mental health concerns based on parents’ observations. The tool consists of a single form with 10 questions covering expressive and receptive language, social–emotional and behavioral aspects, fine and gross motor skills, self-help abilities, school performance, cognitive development, and health issues. Parents respond to each question with ‘yes’, ‘no’, or ‘a little’, and their responses classify the child’s risk level as high, medium, or low. The scoring process considers age-specific predictive markers to identify concerns that may indicate developmental problems. The results are interpreted using an evidence-based algorithm, dividing outcomes into five paths. Path A indicates two or more predictive concerns, suggesting a need for referral, while Path B includes one predictive concern, requiring further screening or observation. Path C involves nonpredictive concerns, typically warranting reassessment or reassurance; Path D highlights parental communication difficulties; and Path E indicates no concerns, requiring only general reassurance or monitoring [8].

Sheel et al. [7] indicated that the PEDS was revalidated and restandardized in 2013 by Glascoe [27], with strong psychometric properties demonstrated through an interrater reliability of 0.95 and a test–retest reliability of 0.88. The tool shows 86% sensitivity and 74% specificity in accurately distinguishing children with developmental delays [28]. It is widely recognized as a promising measure, particularly in low- and middle-income countries (LMIC) [29]. Additionally, the Hindi version of PEDS was evaluated by Sheel et al. [30] in a sample of 466 children aged 4–8 years in India, providing further evidence of its reliability and validity in diverse settings.

### 3.2. Parents’ Evaluation of Developmental Status: Developmental Milestones (PEDS:DM)

Glascoe et al. [31] developed the PEDS:DM (Developmental Milestones), a complementary tool to the PEDS, designed to accurately predict children’s developmental status. The PEDS:DM includes six to eight age-specific items, each addressing a distinct developmental domain such as fine motor, gross motor, expressive and receptive language, self-help, and social–emotional skills, with additional domains like reading and math for older children. The tool is user-friendly, presenting age-appropriate items on a single laminated page with visual stimuli. Parents complete the items in under five minutes using a multiple-choice format. Scoring is straightforward, utilizing a template integrated into the binder. After parents mark their answers, the template is aligned with the questions, and any visible marks indicate unmet milestones. These results are then transferred to a recording form, where developmental progress is visually represented by coloring boxes and drawing lines to map milestones relative to the child’s age [14].

The PEDS:DM employs the same evidence-based decision-making process as the PEDS, categorizing outcomes into different paths based on the results. The tool demonstrates excellent psychometric properties, with an internal consistency of 0.98, test–retest reliability of 0.98, and interrater reliability ranging from 0.82 to 0.96 across subtests. Additionally, it shows a sensitivity of 85% and specificity of 80%, making it a robust measure for identifying developmental delays in children [14].

### 3.3. Procedure

The study was approved by the Human Research Ethics Committee (H8285) to give screening questionnaires to parents of children aged 4–8. Data were collected online between August and December 2021 using Qualtrics. Parents were given validated English and Hindi versions of the measures and could choose the language they preferred for answering the questions [30].

All participants received a participant information sheet outlining the study and the information requested. Schools were contacted first to obtain permission to contact parents for data collection. Schools then approached parents and provided them with a survey link. After giving informed consent, parents completed the demographic questionnaire, PEDS, and PEDS:DM. The total completion time was 15–20 min. Participants could withdraw from the study at any time. To ensure confidentiality, pseudonyms were used for all participants, including parents.

### 3.4. Data Analysis

Researchers analyzed the data using SPSS 18.0. To address the first aim, a Chi-squared test was performed to examine the relationship between general and specific concerns reported by parents of children with TD and DD on the PEDS. The Mann–Whitney U test was used to assess whether the PEDS could effectively differentiate between children with TD and DD. The Kruskal–Wallis test evaluated whether the frequency of concerns reported by parents on the PEDS varied across different age groups. Finally, the ROC curve was used to assess the specificity and sensitivity of both the PEDS and the PEDS:DM.

## 4. Results

### 4.1. Diagnostic Accuracy of the PEDS and the PEDS:DM

The PEDS showed high sensitivity (91%) but low specificity (47%), meaning it was effective at identifying children with DD but less accurate in correctly identifying TD children. In contrast, the PEDS:DM demonstrated poor sensitivity (17%) and specificity (6%) for children in India. Due to the PEDS:DM’s inability to achieve acceptable sensitivity and specificity, the PEDS was utilized to examine the association between parental concerns and developmental outcomes for children with TD and DD.

### 4.2. Parents’ Concerns in General and with Specific Developmental Domains

The study used ordinal levels of measurement in the PEDS tool and applied Ilić et al. [9] chi-square tests of contingency analysis to assess the relationship between parents’ general and specific concerns. The study examined parents’ general concerns in their responses to the first questions on the PEDS form and their specific concerns about developmental milestones in the subsequent questions.

PEDS includes two open-ended questions and eight questions with response options of ‘yes’, ‘no’, and ‘a little’. Certain questions on the PEDS form did not receive any “Yes” answers from participants, so the researchers tabulated only the “no” and “a little” responses. Additionally, they categorized responses for the health and global or cognitive developmental domains as “concerns” or “no concerns” since these were open-ended questions. The researchers presented these responses as “yes” to indicate concerns and “no” to indicate no concerns. The significant relationship between general and specific concerns regarding the PEDS developmental milestones shows that parents who reported general concerns about a developmental milestone were more likely to express the same concern when answering the corresponding specific question on the PEDS form, using “yes” or “a little” as their responses.

### 4.3. Children with Typical Development

The frequency of concerns raised by the parents of children with TD reported that *n* = 218 parents (54%) had no concerns, *n* = 120 parents (29%) had one concern, and *n* = 69 parents (17%) had two or more concerns about their child’s development.

The researchers used Pearson’s chi-square tests of contingency (with α= 0.05) to assess any relationship between general concerns and specific concerns in specific developmental domains on the PEDS test for children with TD. Parents’ general concerns, assessed through the first question of the PEDS form, showed a significant relationship with specific concerns in developmental domains addressed by other questions on the PEDS form. This relationship was statistically significant for expressive language and articulation (*p* = 0.009), behavior (*p* = 0.001), self-help (*p* = 0.020), cognitive development (*p* < 0.001), and health (*p* < 0.001). The results indicated that parents who expressed a general concern about their child’s development were also likely to express similar concerns when answering specific developmental milestone questions (Table 2) [32].

### 4.4. Children with Developmental Disability

The frequency of concerns raised by the parents of children with DD showed that *n* = 5 parents (8%) had no concerns, *n* = 8 parents (13%) had one concern, and *n* = 46 parents (78%) had two or more concerns about their child’s development.

Table 3 presents the relationship between general concerns and specific concerns in specific developmental domains on the PEDS test for children with DD. Parents’ general concerns, reported through the first question of the PEDS form, showed that parents also expressed similar concerns in the areas of expressive language and articulation (*p* = 0.021), fine motor skills (*p* = 0.004), behavior (*p* = 0.027), school (*p* = 0.028), cognitive development (*p* < 0.001), and health (*p* = 0.015). Table 4 compares general and specific concerns on the PEDS test for children with typical development (TD) and children with developmental disabilities (DD) [32].

## 5. Discussion

The study aimed to evaluate the sensitivity and specificity of the PEDS and PEDS:DM tools in classifying 4- to 8-year-old children with TD and DD in India. Additionally, it examined whether parents’ general concerns about their child’s development, as reported through PEDS, were linked to specific developmental concerns indicative of potential developmental issues.

The current findings indicate that while the PEDS demonstrates high sensitivity but low specificity, and the PEDS:DM falls below acceptable levels for both sensitivity and specificity, these tools should not be considered gold standards. Instead, they are best utilized as initial screening tools to identify potential developmental delays in children [33]. Low specificity leads to an increase in false positives, which burdens the healthcare system and heightens parents’ anxiety, expenditure, and stigmatization [23]. Specifically, the low sensitivity and specificity of the PEDS:DM suggests that parents may not fully understand their child’s development [15]. Glascoe [12] noted that overly concerned parents should be seen as vigilant observers who identify behavioral and developmental issues that fall in the grey area between disability and typical development. Clinics and schools across India need to educate parents regarding children’s developmental milestones. Malhi et al. [21] indicated that parental involvement significantly impacts a child’s development, as child-directed verbal engagement markedly influences the early intellectual development of children. Moreover, providing parents access to formal training and educational programs will motivate parents to actively interact and work with their children [34].

In this study, the PEDS demonstrated reasonable test characteristics, making it suitable for use in developmental screening within primary care settings [35]. A unique aspect of the PEDS is its assessment of developmental milestones, where parents’ concerns are categorized as either predictive (significant) or non-predictive (non-significant), allowing children to be classified as high, medium, or low risk for developmental delays. By classifying children based on a plausible risk for the developmental delay instead of labeling them as ‘disabled’ or ‘not disabled’, the tool can help alleviate parents’ anxiety about further assessments. Labeling children is closely linked to stigma, embarrassment, social limitations, and the difficulties of raising a child in a society that devalues disability [36,37]. Moreover, PEDS require a bare minimum of additional material compared to other screening tools, such as the Ages and Stages Questionnaire (ASQ) [38]. High sensitivity and specificity reported in other countries were likely due to the education and knowledge of parents [33].

The study found similar patterns between general concerns and specific developmental domains in both TD and DD children, as assessed by the PEDS tool. Parents of both groups reported a significant relationship between general concerns and specific domains like expressive language, articulation, behavior, cognitive development, and health. Additionally, parents of TD children saw a relationship between general concerns and self-help, while parents of DD children associated general concerns with fine motor skills and school. Interestingly, parents of DD children reported fewer concerns in fine motor skills and school, which might be due to how the questions were worded, especially regarding learning, development, and behavior. However, no significant relationships were found between general concerns and areas like receptive language, fine and gross motor skills, social–emotional learning, and school for TD children. Similarly, for DD children, the link between general concerns and receptive language, gross motor skills, social–emotional learning, and self-help was not significant.

The results from the PEDS form showed that for children with DD, the most significant correlations between general and specific concerns were linked to predictive concerns, while for children with TD, the most significant correlations were linked to non-predictive concerns. Predictive concerns refer to issues related to skills that may signal developmental delay or disability. Participants with high overall PEDS scores typically require referrals for further evaluation. In children with DD, the significant relationship between general and specific concerns mainly involved predictive concerns that indicated developmental delay. In contrast, the significant relationships in children with TD were primarily related to non-predictive concerns, which do not suggest a disability [39]. Parents who reported more than two predictive concerns on developmental milestones such as school, social skills, self-help, and receptive language are often recommended for further testing and early intervention, whereas parents reporting non-predictive concerns are generally advised to seek counseling and follow-up evaluations for their child.

Similar findings have been reported in high-income countries such as Australia [40,41] and the United States [42], as well as in low- and middle-income countries (LMICs) such as Bhutan [43] and Israel [44], where parents of children with both TD and DD expressed concerns across all domains except cognitive development. The current study replicated Ilić et al.’s [9] Serbian study and found a significant correlation in behavior, fine motor skills, and expressive language for children with TD. Furthermore, the results were similar to the findings of Glascoe [6], Soucy et al. [17], Veness et al. [18], Wiggins et al. [19] studies, and contradicted Pinto-Martin et al.’s [20] study.

Mismatches between the purpose of the questions and the real concerns of the parents, as well as the parents’ ignorance of what is developmentally appropriate for their children, are some of the factors that might have affected the current study’s findings [43,45]. For instance, the PEDS form’s Question 1 enquires as to whether parents have any concerns about the development and behavior of their children. Most parents frequently highlighted the effects of online learning during the COVID-19 epidemic on their kids and families rather than voicing any specific concerns. The disparity between the screening tool’s intended use and its actual results may be caused by several reasons, such as culturally specific remarks, inadequate health literacy, and improper developmental expectations [45].

## 6. Limitations and Future Recommendations

The PEDS and PEDS:DM data were gathered online during the COVID-19 pandemic when schools were engaged in remote learning. The findings might not be applicable to all children, as the study only included 4–8-year-olds from private schools in a few North Indian states. This does not represent all of India, which has public schools, 28 states, and many languages. Like in other countries, this study found more boys with DD than girls. Parents’ education levels also varied, with most parents of children with delays only having a high school diploma. This highlights the need for better awareness of developmental milestones and screening in India.

## 7. Conclusions

There is not much research on using PEDS and PEDS:DM in India. This study is helpful because it clarifies how general and specific concerns relate to developmental areas on the PEDS. It found that for children with DD, general concerns were linked to specific areas that predict delays or disabilities. For children with typical development, general concerns were linked to specific areas that do not predict delays. Although the PEDS and PEDS:DM screening tools were not very accurate in identifying children with developmental delays, they still provided valuable preliminary information about how these tools could be used to screen children aged 4–8 in India. This supports introducing parental screening like this into healthcare and preschools in India and possibly other low- and middle-income countries.

## Figures and Tables

**Table 1:** Sociodemographic characteristics of participants.

Demographic Characteristics	TD		DD	
	*n*	%	*n*	%
Gender of the Child Males Females	259 148	64 36	43 16	73 27
Parent Mother Father	276 131	68 32	41 18	69 31
Highest Educational Level Middle School High School Diploma Undergraduate Degree Postgraduate Degree	14 35 21 118 219	3 9 5 29 54	23 4 5 15 12	39 7 9 25 20
Yearly Household Income * <75k 75k–1.5 Lac 1.6–3 Lac 3.1–5 Lac 5.1–10 Lac >10.1 Lac	67 55 42 90 88 65	16 14 10 22 22 16	28 6 9 4 11 1	47 10 15 7 19 2

Note. * A lakh in Indian rupees is equivalent to one thousand US dollars.

**Table 2:** Relationship between general concerns and specific concerns on PEDS test for children with TD.

General Concerns		Specific Concerns		χ^2^
	No	A Little	Yes	
*n* (%)				
Expressive language and articulation				
No	229 (56%)	29 (7%)	1 (<1%)	9.46 *
Yes	115 (28%)	33 (8%)	0	
Receptive language				
No	235 (58%)	24 (6%)	0	0.253
Yes	132 (32%)	16 (4%)	0	
Fine motor skills				
No	244 (60%)	15 (4%)	0	0.208
Yes	141 (34%)	7 (2%)	0	
Gross motor skills				
No	248 (61%)	11 (3%)	0	0.633
Yes	144 (35%)	4 (<1%)	0	
Behavior				
No	228 (56%)	31 (8%)	0	10.45 *
Yes	112 (27%)	36 (9%)	0	
Social–emotional learning				
No	237 (58%)	22 (6%)	0	1.43
Yes	130 (32%)	18 (4%)	0	
Self-help				
No	244 (61%)	15 (3%)	0	5.13 *
Yes	130 (32%)	18 (4%)	0	
School				
No	234 (58%)	25 (6%)	0	3.13
Yes	125 (31%)	23 (5%)	0	
Global or Cognitive Development				
	No	Yes		
No	258 (63%)	1 (<1%)	402.70 *	
Yes	0	148 (37%)		
Health				
No	250 (62%)	9 (2%)	5.19 *	
Yes	135 (33%)	13 (3%)		

Note: * *p* < 0.05. The Table follows the format of a 2 × 3 table. For a “Yes” response, since no responses were received, zero is added to the Table for the specific developmental milestones. “Yes” represents concerns, and “No” represents no concerns.

**Table 3:** Relationship between general concerns and specific concerns on PEDS test for children with DD.

General Concerns		Specific Concerns		χ^2^
	No	A Little	Yes	
*n* (%)				
Expressive language and articulation				
No	22 (37%)	9 (16%)	2 (3%)	8.61 *
Yes	8 (14%)	17 (29%)	1 (1%)	
Receptive language				
No	14 (24%)	13 (22%)	6 (11%)	2.85
Yes	13 (22%)	12 (20%)	1 (1%)	
Fine motor skills				
No	14 (24%)	10 (17%)	9 (15%)	10.37 *
Yes	20 (34%)	6 (10%)	0	
Gross motor skills				
No	20 (34%)	10 (17%)	3 (5%)	0.81
Yes	18 (31%)	7 (12%)	1 (1%)	
Behaviour				
No	17 (29%)	12 (20%)	4 (7%)	7.93 *
Yes	6 (10%)	19 (33%)	1 (1%)	
Social–emotional learning				
No	21 (36%)	09 (15%)	3 (5%)	1.47
Yes	13 (22%)	11 (19%)	2 (3%)	
Self-help				
No	12 (20%)	17 (29%)	4 (7%)	3.79
Yes	13 (22%)	13 (22%)	0	
School				
No	7 (12%)	16 (28%)	10 (17%)	6.48 *
Yes	12 (20%)	12 (20%)	2 (3%)	
Global or Cognitive Development				
	No	Yes		
No	33 (56%)	0	59 *	
Yes	0	26 (44%)		
Health				
No	29 (49%)	4 (7%)	5.57 *	
Yes	16 (27%)	10 (17%)		

Note: * *p* < 0.05. The Table follows the format of a 2 × 3 table. For a “Yes” response, since no responses were received, zero is added to the Table for the specific developmental milestones. “Yes” represents concerns, and “No” represents no concerns.

**Table 4:** Standard deviation comparison between general and specific concerns on PEDS between children with TD and DD.

Developmental Milestone	Relationship Between General and Specific Concerns	
	TD	DD
Expressive language and articulation	9.46 *	8.61 *
Receptive language	0.253	2.85
Fine motor skills	0.208	10.37 *
Gross motor skills	0.633	0.81
Behavior	10.45 *	7.93 *
Social–emotional learning	1.43	1.47
Self-help	5.13 *	3.79
School	3.13	6.48 *
Global or Cognitive Development	402.70 *	59 *
Health	5.19 *	5.57 *

Note: * *p* < 0.05.

## Data Availability

The data are available at https://researchdata.jcu.edu.au/published/b19b543076d211ed9b9a51fb9846a249/ (accessed on 12 December 2023).
